# Refractory seizures in a dialysis patient and a vitamin consigned to oblivion

**DOI:** 10.5414/CNCS111140

**Published:** 2023-09-07

**Authors:** Satish Haridasan, Rakesh Madhyastha, Muriel Ghosn, Fadi Hijazi, Baraa Abduljawad, Mohamed Ibrahim, Rajaish Madhwani

**Affiliations:** 1 Medical Sub-specialties Institute, Department of Nephrology,; 2 Critical care Institute, Cleveland Clinic Abu Dhabi, UAE

**Keywords:** seizures, pyridoxine, levodopa-carbidopa, hemodialysis

## Abstract

Intradialytic breakthrough seizures refractory to multiple classes of antiepileptic medications are not common and can be due to many different reasons. Pyridoxine deficiency is an under-recognized cause of such seizures and frequently missed in clinical practice. Many factors specifically related to dialysis can lead to pyridoxine deficiency and in turn can contribute to refractory seizures. Herein, we report one of the very few cases of intradialytic breakthrough refractory seizures secondary to pyridoxine deficiency recognized in the literature.

## Case report 

A 71-year-old male patient was admitted to our hospital with anemia and sepsis. Five years earlier and after 10 years of progressive symptoms of hand tremors and gait instability, he was diagnosed with Parkinson’s disease. He was started on pramipexole and amantadine, but due to intolerance, he was changed to carbidopa-levodopa which was progressively increased to 25/100 four times daily. Past medical history was also notable for type 2 diabetes mellitus, hypertension, dyslipidemia, and chronic kidney disease for almost 8 years prior to admission. 

Seven months before this current admission, he had post-COVID pneumonia with respiratory failure. He was admitted to another hospital where he was intubated and remained ventilator-dependent. He underwent insertion of a tracheostomy and a percutaneous endoscopic gastrostomy (PEG) for feeding. During that admission, he had episodes of upper gastrointestinal bleeding requiring blood transfusions. He also experienced an episode of seizure with no epileptiform discharges noted on EEG, and he was started on levetiracetam 500 mg once daily. Furthermore, due to the hemodynamic instabilities, his chronic kidney disease progressed. He was hence started on hemodialysis and discharged later to a long-term care facility to continue maintenance hemodialysis 3 times a week through a right internal jugular tunneled dialysis catheter. 

He was transferred to our hospital 6 months later for evaluation and management of persistent methicillin-resistant *Staphylococcus aureus* bacteremia not responding to appropriate antibiotic therapy. Transthoracic echocardiogram done prior to admission had ruled out any vegetations. 

**Figure 1.**
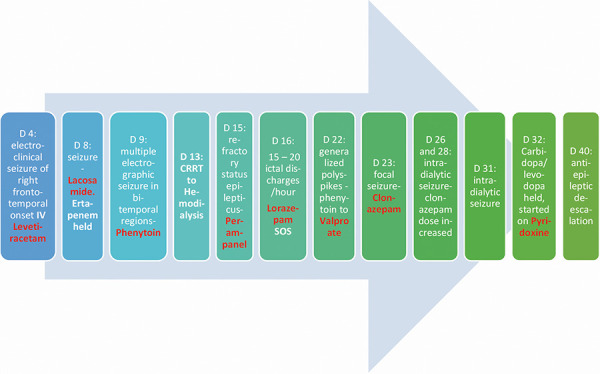
Timeline of seizures and their management.

On admission, he had pallor, pulse rate of 90/min, respiratory rate of 20/min, was bed-bound with Glasgow coma scale (GCS) of 12, on mechanical ventilation through tracheostomy, and had a right chest wound with serous discharge. Laboratory evaluation revealed elevated inflammatory markers with a serum C-reactive protein of 275 mg/L (normal 0 – 4.9 mg/L) and procalcitonin of 3.23 µg/L (normal < 0.04 µg/L). Chest imaging showed bilateral pleural effusions. 24 hours later, he became hemodynamically unstable and was started on inotropes. His tunneled dialysis catheter was replaced with a temporary dialysis catheter, and he was started on continuous renal replacement therapy (CRRT). Four days later, cultures grew extended spectrum β-lactamase (ESBL) *E. coli* in the pleural fluid, *Staphylococcus epidermidis* in the blood, *Candida glabrata* in the chest wound swab, and *Pseudomonas* and *Candida parapsilosis* in the sputum. Right-sided chest tube was inserted, and antibiotic coverage was broadened to ertapenem and fluconazole. He subsequently had a brief episode of facial twitching lasting less than 10 seconds associated with hypertension. The same event recurred after 2 hours for few seconds. EEG recorded electroclinical seizure with a right frontotemporal onset. Levetiracetam was switched from per PEG to intravenous form. He had no seizures for the next 48 hours, leukocytosis and inflammatory markers improved, and he was continued on propofol and fentanyl. Four days later, he had a second episode of seizure activity, and the EEG recorded one tonic-clonic seizure (TCS) with onset in the right hemisphere associated with a versive left head turn. He was loaded with lacosamide followed by maintenance dose. EEG was continued for the next 48 hours and revealed multiple electrographic seizures in bitemporal regions occurring 3 – 4 times per hour, and the doses of levetiracetam and lacosamide were increased. With the possibility of fluconazole and ertapenem lowering seizure threshold, antibiotics were switched to ceftolozane-avibactam. Phenytoin loading dose was then administered followed by maintenance dose. The next 1-week EEG continued to record numerous electrographic seizures. He was considered to have refractory status epilepticus and was started on perampanel. Meanwhile, his hemodynamics had improved and he was switched from CRRT to hemodialysis on the 13^th^ day of admission. Despite the addition of perampanel, he continued to have clinical and electrical seizures with discharges emanating from the left temporal region. Brain MRI showed atrophic and microangiopathic changes. He also underwent a lumbar puncture. Cerebrospinal fluid (CSF) showed normal cell count, normal glucose, marginally increased protein, and was negative for herpes-simplex virus. Unfortunately, the seizures did not subside. Lorazepam as needed, then post-dialysis doses of levetiracetam and lacosamide were added. Phenytoin was eventually switched to valproate on day 22 of admission. Clonazepam was added on day 23, and the dose was subsequently increased (refer to Figure 1 for a detailed timeline of seizures and their management). The refractory seizure episodes were initially attributed to a combination of factors that could have reduced the seizure threshold such as: the use of betalactams, carbapenems, cephalosporins, fentanyl, the presence of sepsis, hypoglycemia and underlying dementia, the worsening brain volume loss and changes of microangiopathy as well as chronic lacunar infarcts seen on brain imaging. However given no improvement in his condition, the dose of hemodialysis was modified by switching to a smaller dialyzer (KoA 1153 to KoA 998), and reducing the blood flow rate (250 mL/min) and the dialysate flow rate (500 mL/min). He again had an intra-dialytic seizure on day 31 of admission which subsided with lorazepam. Extensive laboratory investigations showed no features of ongoing systemic infection, electrolytes or acid base abnormalities, hepatic dysfunction, vasculitis (normal erythrocyte sedimentation rate (ESR), negative antineutrophil cytoplasmic antibody), or thyroid dysfunction. On day 32 of admission, plasma vitamin B6 levels were sent, carbidopa-levodopa was withheld and the patient was empirically started on pyridoxine 40 mg once daily along with thiamine, vitamin B12, and folic acid supplementation. Of note that he was previously maintained in his long-term facility on oral vitamin D, Coenzyme Q (CoQ) and multivitamin tablet of B complex, ascorbic acid, and folic acid. 

There were no further inter- or intradialytic seizure episodes for the next 1 week and his antiepileptics were gradually de-escalated step-wise starting from clonazepam, followed by valproate, perampanel, post-dialytic doses of lacosamide and levetiracetam. A repeat EEG after 2 weeks of pyridoxine showed no epileptiform discharges, and he was resumed on a lower dose of carbidopa-levodopa. Later, reports showed vitamin B6 (PLP) (levels estimated by liquid chromatography-tandem mass spectrometry (LC/MS-MS) low at 8 (14 – 264 nmol/L), with corresponding alkaline phosphatase 178 (40 – 129 units/L), hemoglobin 7 – 8.4 g/dL with macrocytic, normochromic RBCs (hemoglobin was 12.8 g/dL 1 week before admission), transferrin saturation 23%, serum vitamin B12 level 1,476 pmol/L (145 – 569) and 25(OH) vitamin D 97 nmol/mL (50 – 150). After 4 weeks of treatment, vitamin B6 levels had improved to 15 (14 – 264 nmol/L), and he was back to his baseline dose of 500 mg levetiracetam once daily. 

## Discussion 

Intradialytic breakthrough seizures refractory to multiple classes of antiepileptic medications are not commonplace, and vitamin B6 (pyridoxine) deficiency was a lateral thinking in this case. Older age, poor nutrition, chronic dialysis, recent septicemia, switch to high-flux dialyzer, antiepileptics, and levodopa-carbidopa were the risk factors which altogether led to vitamin B6 deficiency. Vitamin B6 is a water-soluble vitamin which is lost during cooking and during dialysis. Deficiency is well known in dialysis patients; plasma levels falling further with high-flux dialyzers and on initiation of erythropoiesis-stimulating agents or phosphate binders [1, 2, 3]. Foods high in vitamin B6, such as wheat bran, avocado, banana, lentils, walnuts, soybean, potatoes, eggs, meat, fish, cheese, and milk, are also often restricted in the hemodialysis population owing to their potassium and phosphate contents. 

Carbidopa is a hydrazine derivate, which binds covalently and irreversibly in 1 : 1 ratio to pyridoxal-5’-phosphate (PLP), blocking L-DOPA decarboxylation, and chronic treatment with carbidopa produces a long-term systemic vitamin B6 depletion or functional deficiency [4]. 

Pyridoxine and PLP deficiency impair the synthesis of GABA from glutamate and lower the seizure threshold. There is high prevalence of pyridoxine deficiency in status epilepticus, and deficiency is also found to impair seizure control in adult epileptics [5, 6, 7]. Successful response to vitamin B6 replacement has been seen in separate cases of pharmacorefractory seizures including patients on maintenance hemodialysis [8, 9, 12]. A maintenance dose less than 100 mg/day was found to be safe by Chaudary et al.’s retrospective study [10] and besides being antiseizure, pyridoxine has pleotropic effects benefiting cognition, erythropoiesis, and microvascular integrity. 

Possibly due to under-recognition, case reports of pyridoxine deficiency as a risk factor for pharmacorefractory seizures in adults are sparse [8, 9, 11]. The use of antiepileptics and carbidopa in patients on maintenance hemodialysis, as in our case, can exponentially increase the risk for deficiency. Hence, pyridoxine deficiency should always be considered in similar scenarios and supplementation initiated when suspicion is high. 

## Funding 

The authors did not receive support from any organization for the submitted work. 

## Conflict of interest 

The authors of this manuscript declare no conflict of interest.
